# Design of Urban Garden Landscape Visualization System Based on GIS and Remote Sensing Technology

**DOI:** 10.1155/2022/9592376

**Published:** 2022-09-22

**Authors:** Wenpeng Zhang

**Affiliations:** ^1^School of Financial Technology, Shanghai Lixin University of Accounting and Finance, Shanghai 201209, China; ^2^University Kuala Lumpur Business School, University Kuala Lumpur, Wisma Yayasan, Selangor 50300, Kuala Lumpur, Malaysia

## Abstract

Urban green ecological space is an important manifestation of the environmental characteristics of a green city. The research results show that the urban green ecological space has obvious cooling and humidity effects, which are very important for reducing the urban heat island effect. Remote sensing technology describes the slow-release effects of urban green parks in different seasons from the two perspectives of thermal slow-release intensity and thermal slow-release distance. In this paper, UAV remote sensing is used to extract the internal and external factors of the urban green environment characteristics and to identify the main factors that affect the slow-release heat effect and seasonal changes of the urban green environment. In addition, it analyzes the factors that affect the urban environmental temperature within the environmental temperature slow-release range of urban green space, establishes a model to predict the environmental temperature within the thermal slow-release range outside the park, and realizes the largest thermal slow release in the urban greening ecological space. These are new technologies created in the context of digitization, which include image understanding and synthesis, which involve the use of computer graphics and image processing technology to convert data into graphics or images displayed on the screen to achieve an interactive process.

## 1. Introduction

This article mainly studies the visualization of urban landscape. The main research object is the shape of urban landscape groups or individuals. Then, using the rapid development of virtual reality technology in recent years, the growth process of the landscape of the research object in the three-dimensional space is produced on the computer. Urban landscape visualization has become the main focus of this article. Landscape visualization refers to the use of virtual reality technology to reproduce the growth process of the landscape in a three-dimensional space on the computer to study the shape and structure of the landscape [1]. Visualization is mainly through observation and measurement, using computer software to set the landscape growth parameters, and then adjusting based on experience to obtain the ideal landscape effect [2]. In the later work, it is also necessary to change the texture and color of the landscape organs through the graphics library to make the modeling of landscape growth clearer. In the urban garden landscape, three-dimensional modeling and visualization have achieved concrete results, mainly including landscape matrix and landscape model [3]. However, the effectiveness in landscape design is now expressed in a two-dimensional, qualitative, and static form. This article uses landscape visualization in landscape design for the first time to create a dynamic display landscape in a time-based design. The landscape performance achieves a four-dimensional effect [4]. The design is also more intuitive, rich, and practical. Most software currently used in garden design cannot show landscape visualization. After screening, this article chose the GreenLab visualization software provided by the Institute of Automation to visualize a single landscape [5]. If you want to realize the application of landscape visualization in garden design, first of all, you must have a powerful database. In the next stage, combined with the garden design made during the internship, landscape visualization is introduced into the static 3D landscape, creating new garden landscape efficiency and making the garden landscape effect more dynamic, flexible, and creative [6]. Then, combined with remote sensing technology to analyze the characteristics of the green city environment, it is concluded that the heat release effect of the urban green ecological space has seasonal changes [7]. In summer and winter, the average temperature in a certain park is lower than the temperature outside the park. As the distance from the park boundary increases, the temperature also rises, which also proves that the green air of the city affects the city's heat-releasing effect [8]. The average temperature in the park at night in winter is higher than the surrounding environment of the city, reflecting the “warm island effect” of the city's green environment. From summer to winter, the average slow heat release (CEI) intensity of the park decreased from 3.63°C to 2.02°C, and the average slow heat release (SCE) distance decreased from 246 m to 172 m.

## 2. Materials and Methods

### 2.1. Theoretical Basis

The sequence of data processing is radiometric calibration, atmospheric correction, geometric correction, and ortho correction. This article mainly studies radiation calibration and atmospheric correction. Geometric correction can ensure that the quality of satellite images is consistent with their original position on the Earth's surface. In this study, the metadata provided by Landsat 8 imagery were used for correction. In addition, the purpose of radiometric correction is to provide data with pixel values related to surface backscatter. The radiometric data are calibrated for backscatter coefficients, atmospheric correction is performed to improve data quality, and FLAASH (fast line of sight) is used to perform hypercube spectral analysis. FLAASH can analyze a wide range of visible light, multispectral and hyperspectral. Use remote sensing image processing tools to combine panchromatic bands to achieve higher spatial resolution (region extraction accuracy will be comparable to that of multispectral bands, and there is no need for later merging).

Affected by objective factors limited by the satellite direction and altitude, the image obtained by the sensor will cause geometric distortion, which must be corrected using the proposed correction model; geometric correction includes the following concepts:

  Geocoding: rectify the image to a single standard coordinate system.
  Geotagging: use multiple checkpoints to correct the geographic coordinates of the image.
  Image registration: an image (reference image) in an area is calibrated with another image.

The general steps for correcting the geometric accuracy of an image are as follows. This is the most important step for correcting the geometry. Checkpoints can be selected from the topographic map as a reference, can also be obtained from a field survey, or can be obtained from a calibration image. The selected control points have the following characteristics: remote sensing data have obvious characteristics, such as reservoirs, construction land, rivers, and so on; the ground objects of the control points will not change with time.

### 2.2. Research Methods

In this study, Thermochron iButtons sensors are used to collect temperature and humidity inside and outside the urban green environment.

Mean square error:

(1) $$\begin{array}{c} RMSE=\sqrt{\frac{\overset{n}{\underset{i=1}{\sum }}{\left(x_{1,t}-x_{2,t}\right)}^{2}}{n}}\text{.} \end{array}$$

*X *_1,*t*_ and *X*_2,*t*_ represent the measurement data of iButtons and weather station, respectively. Correlation analysis:

(2) $$\begin{array}{c} r=\frac{\sum \left(X_{i}-\bar{X}\right)\left(Y_{i}-\bar{Y}\right)}{\sqrt{\sum {\left(X_{i}-\bar{X}\right)}^{2}\sum {\left(Y_{i}-\bar{Y}\right)}^{2}}}\text{.} \end{array}$$

*X *_*i*_ and *Y*_*i*_ represent the observed values of iButtons and weather stations, respectively, while $\bar{X}$ and *Y* represent the average values of iButtons and weather station sample values, respectively. Significant difference test:

(3) $$\begin{array}{c} t=\frac{\bar{X_{1}}-\bar{X_{2}}}{\sqrt{\left(\left(n_{1}-1\right)S_{1}^{2}+\left(n_{2}-1\right)S_{2}^{2}/\left(n_{1}+n_{2}-2\right)\right)\left(1/n_{1}+1/n_{2}\right)}}\text{.} \end{array}$$

*S*_1_^2^ and *S*_2_^2^ are the variances of the measured data of iButtons and the weather station, *n*_1_ and *n*_2_ are the sample sizes, and $\bar{X_{1}}$ and $\bar{X_{2}}$ represent the average values, respectively. Thermal release strength:

(4) $$\begin{array}{c} CEI=\frac{2b^{3}+\left(2b^{2}-6ac\right)*\sqrt{b^{2}-3ac}-9abc}{27a^{2}}\text{.} \end{array}$$

CEI is the thermal sustained release strength of the study area. Thermal release impact distance:

(5) $$\begin{array}{c} SCE=\frac{-b-\sqrt{b^{2}-3ac}}{3a}\text{.} \end{array}$$

SCE is the thermal slow-release influence distance in the study area; *a*, *b*, and *c* are the coefficients of the cubic term, quadratic term, and first term in the cubic polynomial, respectively.

## 3. Results

### 3.1. Environmental Temperature Characteristics of Green Cities

**Figure 1:**
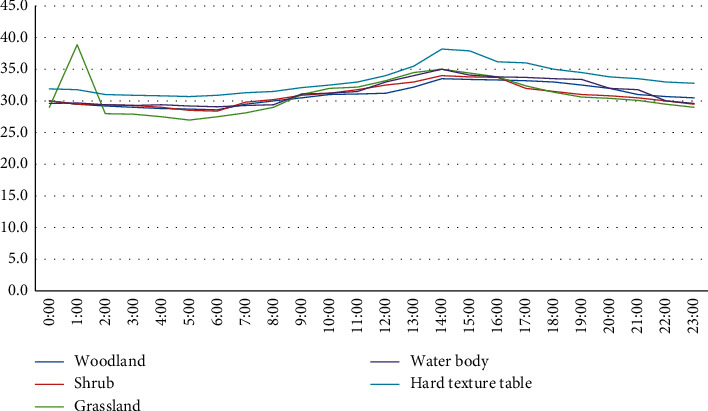
Diurnal changes in air temperature of five underlying surfaces in summer.

Figure 1 shows the temperature change characteristics of five planting surfaces in a park, including forests, shrubs, grasslands, ponds, and hard surfaces in summer. It can be seen from the figure that the daily temperature characteristics of the five underlying surfaces in summer are in an inverted U-shaped distribution, with obvious troughs (05:00) and peaks (14:00). After sunrise (6 o'clock in the morning), the average temperature of the five surfaces of forests, shrubs, grasses, ponds, and hard surfaces will increase over time. From 12:00 to 14:00 in the afternoon, the temperature of the underlying surface is the highest.

From the temperature difference of the following surface types, Table 1 shows the result of Student's *t*-test on the temperature of the five main surfaces in summer. The results show that there is a difference in the average summer temperature between the hard ground, the water bod,y and the other three planting surface(*P* < 0.05).

**Figure 2:**
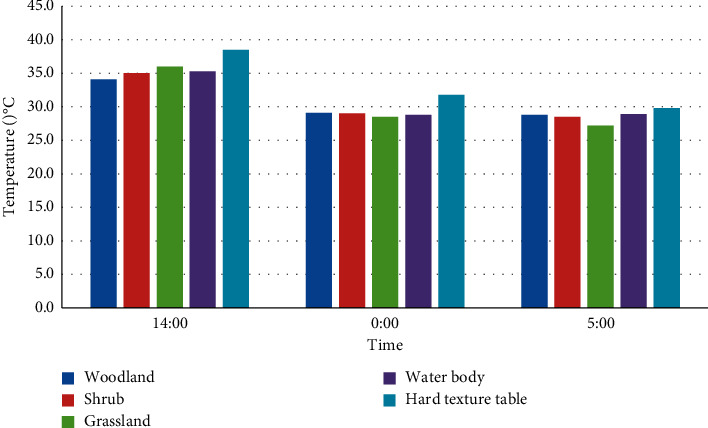
The air temperature difference of the underlying surface during the three periods of noon, midnight, and sunrise in summer.

For this, this study compares the temperature difference between the five main surfaces of a park during the afternoon (14:00), midnight (00:00), and sunrise (05:00) (Figure 2).

**Figure 3:**
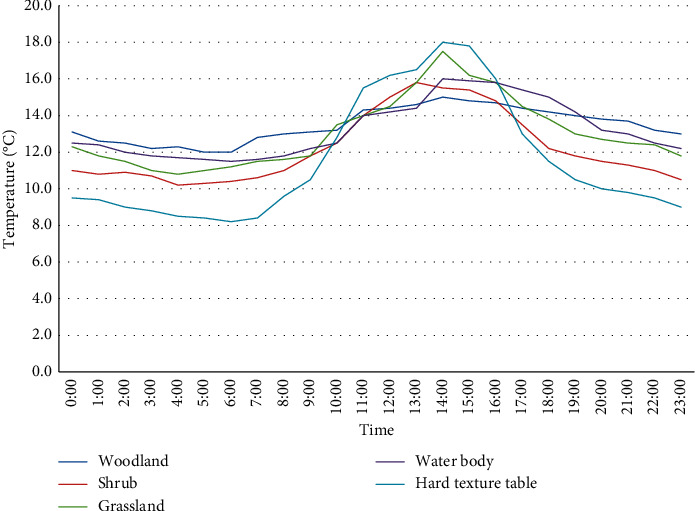
Diurnal changes in air temperature of five underlying surfaces in winter.

Figure 3 shows the daily temperature changes of the five underlying surfaces in the winter park. It can be seen from the figure that the diurnal variation characteristics of the five surfaces in winter are in an inverted U-shaped distribution, but the degree of line oscillation is not as strong as in summer, and there is only an obvious peak in winter (14:00).

Table 2 shows Student's *t*-test test results of five underlying surface temperatures in winter. From the temperature difference of the underlying surface types, the average temperature of the five underlying surfaces (woodland, shrubs, grassland, water body, and hard surface) in winter all have certain differences (*P* < 0.05). Among them, the *P*values between vegetation (woodland and shrubs, shrubs, and grassland) are all less than 0.05, which indicates that there is a big difference in temperature between woodland and shrubs, shrubs and grassland in winter.

This study compares the temperature difference between five underlying surfaces during the day (14:00), midnight (00:00), and before sunrise (05:00).

### 3.2. Environmental Comfort Characteristics of Green Cities

Environmental comfort is a manifestation of emotional well-being, and it is the satisfaction of psychological and physical problems to the environment. With the gradual increase in the intensity of urban tropical islands, it will have different levels of impact on the living environment of urban people. A large number of studies have confirmed that urban green spaces have the effects of cooling and humidification. The comfort of green cities is also judged by two aspects, such as climate and environment, which can directly affect human senses, and can also directly complete perceptual evaluation by senses. The temperature of the urban environment is discussed above. This section analyzes the impact of different summer trees on environmental comfort under natural conditions. This paper selects forests, shrubs, and grasslands to study, calculates the individual comfort levels of different types of underground and outdoor areas in the park, and compares them, and improves the difference of human comfort through different environmental planning.

Table 3 calculates the discomfort index of different types of underlying surfaces at different times in summer. It can be seen from the table that the average discomfort rate of forests, shrubs, and grassland is less than 27, and only a few people have discomfort. This is the best place for activities. The daily average values of discomfort index of control points and hard surfaces are both greater than 28.

The thermal comfort improvement degree (HCI) of various underlying surface types in a park in summer is calculated. The picture shows that different time periods and different types of surfaces have different degrees of improvement in water body comfort. In summer, the comfort improvement (HCI) of forests, shrubs, and water bodies is the most important one. The improvement of thermal comfort is 2.68–4.35, and the average improvement is 2.68.

Table 4 shows the relationship between the temperature, discomfort index, and structural characteristics of each surface that exist in summer and winter. It can be seen from the table that vegetation temperature (forest, shrubs, and grassland) is negatively correlated with area and three-dimensional green number and positively correlated with sky visibility coefficient (*P* < 0.05), while hard surface temperature and sky visibility coefficient are also positively correlated (*P* < 0.05).

### 3.3. Visualization Process of Urban Garden Landscape

Three-dimensional visualization is a tool used to display and explain the characteristics of soil geological phenomena and is widely used in geological and physical research. 3D visualization is different from various modeling technologies. It uses a form of expression that uses model data to visualize and understand things. Three-dimensional visualization technology can model the mechanical production process, reveal the reliability of data, and find the need to solve current problems. Technical support for analytical modeling and decision making bridges communication and collaboration between various disciplines. The realization of 3D visualization is mainly divided into two categories: construction model and texture display.

**Figure 4:**
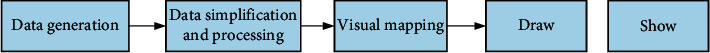
3D spatial data visualization process.

Three-dimensional visualization, or three-dimensional cartography, is a major function of geographic information systems. The research field of three-dimensional imaging is in the environment of three-dimensional space, which contains a wide variety of shapes, complexities and varieties, and spaces of different sizes. The visualization process of three-dimensional spatial data is shown in Figure 4.

*Generated Data*. This is a combination of three-dimensional objects in space using abstract data and images to perform visual effects on them. For example, field data collection uses digital altitude models for aerial photography. Digital elevation models are created by inserting and fitting polynomials to multiple discrete elevation points.

*Simplification and Data Processing*. Achieving 3D visualization requires the maximum possible accuracy and precision of the model while ensuring system speed and visualization effects and configuring appropriate system software and hardware to respond to the real situation of large 3D spatial data, such as LOD or detailed model level.

*Drawing*. In order to better improve the performance of real 3D objects in the 2D plane, it is necessary to convert various data and visually mapped object data into graphics that can be viewed on a computer. Calculate the color of each pixel on the video screen, and the color value of each pixel can be displayed on the screen.

### 3.4. Construction of Urban Garden Landscape Visualization System

**Figure 5:**
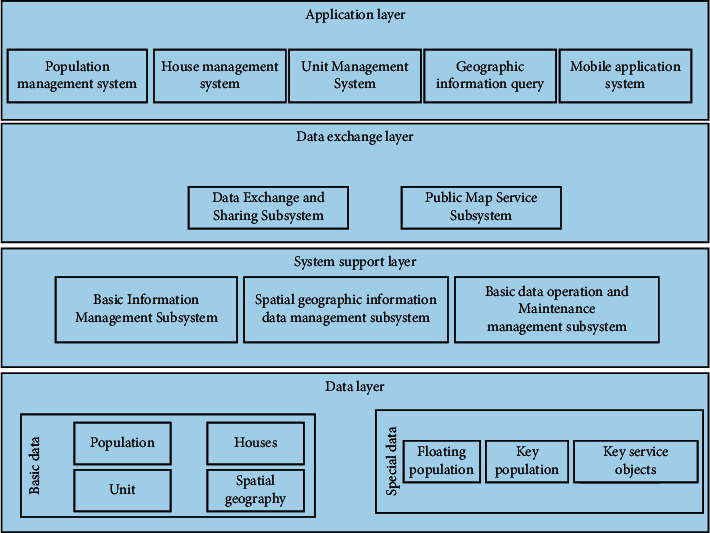
The overall frame structure of the system.

The overall design of the 3D visual city system in a certain area adopts the traditional structure of CIS, that is, the client structure. This structure can use the hardware advantages of both parties to provide sufficient complete client and server tasks, thereby reducing development costs and improving operational efficiency. Considering a large amount of data processing and system integration, the organization of three-dimensional scenes, and a large amount of data release, the advantage is to avoid direct contact between customers and data, which affects system performance. The system is shown in Figure 5.

Skylin TerraSuit generally includes three product types: TerraBuilder, TerraExplorer Pro, and TerraGate. The three types of products are integrated to manage different geospatial data and quickly distribute them to users in the office, at home, or on-site. The 3D modeling of modern organization and urban data management methods usually uses two methods: file format and object-oriented relational spatial database. Traditional geometry and texture data storage libraries are stored separately, forcing a large amount of spatial information and related columns to generate redundant information of index relations, which is not conducive to data management, maintenance, and update. The object-oriented relational spatial database management method uses aggregate data and related texture data, stored in a spatial model, and each three-dimensional information is regarded as an entity with the same spatial information and attributes. This not only simplifies data query and retrieval but also improves the security of database management and retrieval of historical data. The data management model of this project uses the corresponding tools on the ArcGIS platform to convert 3ds into multibatch data types and connects to the Oracle database through the ArcSDE spatial data engine to realize data addition, deletion, and modification operations, and finally realizes the management and maintenance of the 3D database. ArcSDE can capture, retrieve, update, and manage large amounts of data, which can improve system operating efficiency and manage model data more effectively.

An important data framework for 3D terrain visualization includes 3D terrain data. This is a model of the terrain surface, created by technologies such as terrain modeling and texture mapping. It does not include landscape models and 2D vector data. As the basis for creating 3D scenes, they only need to use Skyline TerraBuilder to combine digital high-resolution model data and image texture data with remote sensing processing.

System implementation is the process of transforming the design results into a real controllable system, that is, transforming the logical model and physical model of the design phase into practical applications, and it is necessary to use a programming language to build a system that people can use. First, the program is developed using one or more programming languages, and then the program is tested and debugged. The development and realization of the 3D visualization system has the characteristics of a wide variety of data, a large amount of data, and real-time display requirements. Therefore, there are specific requirements for the software and hardware environment and operating mode of the development and operation of the system.

The first is the preparatory work, including data collection, plan formulation, staffing, and project allocation. Then, arrange for staff to collect textures on site. The photographer must sort the data on the same day and send it back to the office team for texture processing. Secondly, according to the data obtained, 3D models are made using 3ds Max, and the 3D models required by the urban area are made. Using preprocessed data and texture data collection, 3D modeling is performed on a 1 : 500 topographic map according to the outline of the building, and the texture data processed at the same time are applied to the map to export the built-in 3D model.

## 4. Discussion

### 4.1. Analysis of Environmental Characteristics of Green Cities

This paper analyzes the thermal environment characteristics of five major garden surface types. Studies have shown that the surface temperature of different city parks is different in summer and winter. The five surfaces have different air temperatures, and the daily temperature fluctuation characteristics are in an inverted U-shaped distribution [9]. The fluctuation range in summer is definitely stronger than that in winter, and the intensity of fluctuation during the day is stronger than that at night. In summer and winter, when the surrounding temperature rises, the temperature of each underlying surface rises. At 14:00 in the afternoon, the daily average temperature of each surface was the highest; after that, the temperature of the underlying surface dropped slightly. As for the first time the sun comes out (5 o'clock in the morning), the temperature of each surface is the lowest in the day. In the temperature difference of the underlying surface, the temperature difference of the five underlying surfaces in summer is inversely proportional to the temperature difference in winter, and the temperature difference in summer is definitely greater than that in winter. In summer, the temperature difference between the five most obvious surfaces—up to 4.2°C, and the lowest temperature at midnight in summer—is only 2.7°C. Different from summer, the minimum temperature difference between the underlying surface in winter is 2.7°C, and at night, the temperature difference on the underlying surface increases with the passage of time.

In summer, the diurnal variation characteristics of the thermal slow-release intensity (CEI) of the five underlying surfaces generally present a “U-shaped distribution,” with an obvious peak (05:00) and a trough (14:00). During the summer day, the woodland, shrubs, and water bodies in the park have a certain degree of heat release strength, and the temperature of the grass and hard surface is higher than the temperature of the external reference point of the park, which becomes the heat source inside the park. In summer, this paper calculates the diurnal variation characteristics of the discomfort index from the external control points of the park and the underlying surface of the five parks [10, 11]. It can be seen that the discomfort index of the five underlying surfaces in the park increased first and then decreased, showing a single-peak curve, reaching a peak in the summer afternoon; therefore, the discomfort of the human body gradually decreases, and the discomfort is the lowest when the sun rises. Compared with the external control point of the park, vegetation such as forests, shrubs, and ponds can effectively improve the thermal comfort of the human body. The improvement rate of human comfort is 2.68–4.37, with an average of 2.67. Although the improvement of the ranch during the high temperature (12:00∼14:00) period is limited, the ranch still has a better chance to improve the thermal comfort in other periods. For cities with high temperature and high humidity in summer, it is recommended to increase the underlying surface area of forests, shrubs, and ponds to reduce the harmful effects of high temperature.

### 4.2. Requirements and Principles of Urban Garden Landscape Visualization System Construction

In combination with the wide spread of regional improvement and innovative social governance, and in accordance with the requirements of collective integration and effective management, people should formulate and improve the concept, and strengthen and create innovative social government information systems in advanced urban areas at home and abroad. People promise to create a comprehensive, multifunctional, and specific regional urban landscape visualization system based on practical work, according to the requirements of “internal managers,” with electronic maps as operators and 3D geographic information technology as support. This can directly and clearly reflect the relationship between regional population, buildings, units, events, organizations, and spatial geography, and understand the relationship between population, housing, and units [12]. It also has the functions of searching and locating subject databases, statistical analysis and providing relevant service management solutions [12]. It also has functions such as searching and locating the subject database, statistical analysis and providing related service management solutions.

Demand analysis is the analysis of the needs of users who use the system. Developers should strive to ensure that the developed system can meet the needs of users. With the help of demand analysis, developers can determine in advance the type of data required by the system, plan how to manage the system database, develop functional modules, and so on [13]. The 3D visualization system in this area is based on the city's remote sensing images, 2D data, and 3D model data. People use GIS technology, combined with computer graphics, 3D visualization, database technology, etc., through the creation of accurate 3D building models and virtual reality technology, to achieve interactive 2D and 3D geographic information query, 3D data roaming, information retrieval and other basic functions, to achieve 3D spatial analysis and mobile terminal management. People combine the Internet and mobile communication network to build urban information service system [14]. The specific performance is as follows:

1. Complete basic data informatization construction.
2. Based on housing, the system establishes three major databases of population, housing, and units and forms direct links with information such as roads, communities, networks, buildings, units, floors, and households. The combination of spatial geographic information realizes functions such as population information query, housing information query, unit information query, accurate population address positioning, and regional demographic analysis.
3. 3D model effect display with artistic characteristics.
4. The system uses standardized, advanced programming and real-time rendering technology to create a three-dimensional display of lifelike urban effects. Displaying and exploring three-dimensional information intuitively and quickly determines people's views on the stage.
5. Efficient operation and management of massive data.
6. The two-dimensional vector data and city model data contained in the system have a large amount of data. Professional technical means must be used to quickly view the massive modeling data.
7. Wide range of practical applications, convenient for the management of various departments.
8. Provide authoritative, fresh, comprehensive, and efficient geographic information services in accordance with the requirements of “one stop.” In accordance with relevant technical standards and specifications, with the “command platform” as the main body, it provides convenient and efficient system services for decision-making departments to use, which is convenient for all departments.
9. Develop a mobile terminal management system to ensure the authenticity of information.
10. In the mobile PDA of the smart terminal, create basic data entry functions, modify query functions, and support comprehensive statistical analysis of some data, so that community or field staff can enter the population, housing, and unit data into the space for query and modification. It can classify events and components in time and upload them to district, road, and community management platforms from different perspectives.

### 4.3. Analysis of the Importance of Urban Garden Landscape Visualization

The current garden environment is highly valued. In real estate transactions, people do not usually buy houses in this way. The ecological landscape of the community is gradually becoming the main factor that forces people to buy houses [15]. When people buy a house, they also need to look at a good ecological environment. Therefore, the community not only needs a good living environment and beautification but also hopes that the place of residence has a fresh and lively atmosphere.

Community greening is the main component of the community environment. Generally speaking, this applies to the management of residential buildings, public buildings, and greening outside the residential area, garden buildings and garden sketches, or roads within the residential area, or land for leisure and entertainment provided for residents. The green area is more in line with the lives of residents and better serves residents. Public green space is a good place for residents to carry out daily outdoor activities; green space creates necessary ventilation and lighting, and the visual space between residential buildings is usually green. This can effectively improve the ecological environment of the residential area; thanks to landscaping and construction cooperation, the open space of the residential area has undergone many changes, forming a pleasant and typical residential area landscape environment.

The most important point of beautifying the community is the coordination with the environment, not just the requirement for the amount of greenery. This kind of coordination is not only something that some grasses, flowers, trees, and shrubs can do, but also a higher level of greening should be achieved by improving the quality of indoor greening. The greening of high-quality houses emphasizes the relationship between the park landscape and life and culture. Under the premise of the combination of garden landscape, architecture, garden waterscape, paving, garden trails, etc., make full use and protection of natural resources to create a cultural landscape and a lofty ecological living environment.

*Environmental Protection Function*. Since the main element of the residential landscape is the landscape, landscaping can not only reduce dust, purify the air, absorb sound, and protect the environment but also help improve the climate of the residential area, effectively absorb the heat of the sun, adjust the temperature accordingly, and reduce the wind speed at the same time. In addition, when there is no wind in summer, there is a certain temperature difference between the indoor and outdoor environments of the living space, which can increase the air exchange and the formation of wind.

*Aesthetic Function*. Colorful green communities, seasonal urban landscapes, some decorative buildings, and various landscape decorations not only enhance the appearance of the home environment and residential area, but also make the residential community more lively and interesting.

## 5. Conclusion

The thermal slow-release effect of urban green space is the result of the comprehensive effects of different underlying surfaces on the environmental green space. The area ratio and spatial structure characteristics of different underlying surface types have an important impact on the environmental thermal slow release, and the greening effect and human comfort also have significant influence. This paper then conducts research on landscape visualization, showing the growth process of using virtual reality technology in landscape visualization space. Visualization is mainly through observation and measurement; visualization software adjusts landscape growth parameters and then adjusts based on experience to achieve the expected landscape growth effect. In the later work, it is also necessary to change the texture and color of the landscape organs through the graphics library to make the modeling of landscape growth clearer. Then, this paper realizes the three-dimensional visualization design of the urban landscape in the research area based on specific research projects. Combining theory with practice, it studies the three-dimensional terrain dataset and three-dimensional model data in depth and summarizes the construction requirements and principles of the urban garden landscape visualization system based on the actual needs of the project. Finally, a three-dimensional visualization system was established on the Skyline platform combined with the database and ArcGIS Engine.

## Figures and Tables

**Table 1:** The temperature difference of five underlying surfaces in summer (Student's *t*-test test results).

Underlying surface type	Woodland	Shrub	Grassland	Water body	Hard ground
Woodland	—	0.197	0.87	0.018	0.001
Shrub	0.197	—	0.205	0.012	0.001
Grassland	0.87	0.205	—	0.015	0.001
Water body	0.018	0.012	0.015	—	0.001
Hard texture table	0.001	0.001	0.001	0.001	—

**Table 2:** Student's *t*-test test results of the temperature difference of five underlying surfaces in winter.

Underlying surface type	Woodland	Shrub	Grassland	Water body	Hard texture table
Woodland	—	0.001	0.137	0.116	0.003
Shrub	0.001	—	0.001	0.001	0.016
Grassland	0.137	0.205	—	0.015	0.001
Water body	0.116	0.001	0.385	—	0.002
Hard texture table	0.003	0.016	0.001	0.002	—

**Table 3:** Discomfort levels of five underlying surfaces and control points at different moments.

Time	0:00	2:00	4:00	6:00	8:00	10:00	12:00	14:00	16:00	18:00	20:00	22:00	Daily average
Woodland	26.26	25.67	25.06	24.83	27.08	28.38	28.96	29.19	29.16	28.41	28.19	27.32	27.37
Shrub	27.09	26.33	25.53	25.25	26.78	28.46	29.28	30.14	30.61	29.39	28.76	28.07	27.97
Grassland	26.08	25.48	25.23	24.68	26.12	29.01	30.86	31.17	30.88	29.57	28.78	27.52	27.94
Water body	26.98	26.78	26.68	27.24	27.45	29.43	30.45	30.85	30.27	29.47	28.28	26.63	28.37
Hard texture table	27.81	27.75	27.48	28.06	28.82	30.76	31.74	33.04	31.96	30.88	29.95	29.04	29.77
Control point	28.82	29.08	28.97	30.46	30.76	31.55	32.69	33.54	31.74	30.74	30.68	29.88	30.74

**Table 4:** Correlation between air temperature, discomfort index, and structural characteristics in summer and winter.

Factor	Underlying surface type	Structural feature factor		
		Area	Three-dimensional green quantity (3D-GQ)	Sky visibility factor (SVF)
Air temperature (summer)	Woodland	−0.412	−0.77	0.526
	Shrub	−0.376	−0.577	0.758
	Grassland	−0.234	−0.385	0.792
	Hard texture table	0.396	—	0.718

Discomfort index (summer)	Woodland	−0.523	−0.828	0.642
	Shrub	−0.446	−0.494	0.744
	Grassland	−0.28	−0.366	0.89
	Hard texture table	0.242	—	0.922

Air temperature (winter)	Woodland	−0.365	−0.378	0.473
	Shrub	−0.266	−0.462	0.514
	Grassland	−0.136	−0.214	0.411
	Hard texture table	0.359	—	0.569

## Data Availability

The data used to support the findings of this study are available from the corresponding author upon request.
